# Occupational exposure to HIV in a developing country: assessing knowledge and attitude of healthcare professional before and after an awareness symposium

**DOI:** 10.1186/s13104-018-3231-y

**Published:** 2018-02-15

**Authors:** Samina Ismail, Safia Awan, Rubaba Naeem, Sarfraz Siddiqui, Badar Afzal, Bushra Jamil, Uzma Rahim Khan

**Affiliations:** 1 Department of Anaesthesiology, Aga Khan University, Karachi, Pakistan; 2 Department of Emergency Medicine, Aga Khan University, Karachi, Pakistan; 3 Department of Medicine, Aga Khan University, Karachi, Pakistan

**Keywords:** Health care providers, Occupational exposure, Human immunodeficiency virus, Post exposure prophylaxis, Continuing medical education, Knowledge, Attitude, Prevention

## Abstract

**Objective:**

Health care providers (HCPs) are at risk of occupational exposure to HIV infection. In developing world these exposure occur due to general lack of awareness, education and structured training of HCPs. The objective of the study was to asses if continuing medical education symposium can be used as an effective educational tool to improve attitude, awareness and knowledge regarding occupational exposure to HIV infection. This quasi-experimental study was conducted among HCPs from Karachi, Pakistan. After assessing the baseline knowledge, awareness, and attitude by means of pretest; HCPs were reassessed with posttest after an education symposium on occupational exposure to HIV infection.

**Results:**

Among 364 participating HCPs, 14.2% had previous training on post exposure prophylaxis. There was an overall statistically significant (P value < 0.001) improvement in the attitude of the participants. A statistically positive improvement in the number of participants giving correct answer was observed in 9 out of 11 questions (P value < 0.001). The mean score of participants’ knowledge before intervention was 6.44 ± 1.84, which improved to 8.82 ± 2.17. Along with the increase in knowledge, a positive change in the attitude regarding safety against HIV was observed after the education symposium.

## Introduction

Health care providers (HCPs) are at risk of occupational exposure to human immunodeficiency virus (HIV) infection [1]. The World Health Organization estimates the global burden of HIV infection from occupational exposure to be 2.5% among HCPs [2]. It is estimated that 90% of these occupational exposures occur in the developing world due to general lack of awareness, education and structured training regarding prevention and measures to be taken in case of accidental exposure to HIV infection [3].

Post exposure prophylaxis (PEP) includes measures that are taken after getting exposed to HIV infection. PEP includes first aid, counseling, risk assessment, relevant laboratory investigations and short term treatment with antiretroviral drugs for 28 days, along with follow-up and evaluation [4]. Literature shows that there is information gap among HCPs regarding PEP [4–6].

Even though Pakistan has a low burden of HIV infection, with an estimated 85,000 people or 0.1% of the adult population living with HIV, there is a considerable threat of HIV spread across the country [7]. In Pakistan, preventive measures like continuous surveillance and education related to HIV are still inadequate, mainly because of low healthcare budget [8].

Therefore in developing countries like Pakistan, where there is already lack of educational initiatives for HCPs, it is highly important to find a way to provide basic level of knowledge pertaining to occupational exposure to large number of HCPs in a short period of time. Pakistan Medical and Dental Council (PMDC) have developed standards and guideline on use of continuing medical education (CME) as a mandatory requirement for the renewal of license to practice medicine. Applying CME symposium as an effective educational tool to provide knowledge regarding prevention of occupational exposure to HIV and PEP, still needs to be determined.

The objective of the study was to evaluate the effectiveness of this education symposium by (1) assessing baseline knowledge and attitude pertaining to prevention of occupational exposure of HIV and PEP among health care providers from Karachi, Pakistan (2) re-assessing the knowledge and attitude after providing basic level of knowledge through awareness symposium on HIV addressing occupational exposure of HIV and PEP (3) comparing the results of pre and post assessments.

## Main text

### Materials and methods

After hospital ethics committee approval, this Quasi-experimental study was conducted among HCPs in February, 2016 at a tertiary care university hospital. An educational CME symposium accredited by the PMDC and the American Association of Continuing Professional Education was formulated. The symposium and its objectives were advertised through flyers and banners, which were sent manually and by emails to concerned departments of different hospitals of Karachi. The HCPs included in the study were physicians, nurses and technicians from number of specialties including anesthesiology, surgery and emergency medicine. We choose the relevant specialties to accommodate HCPs who are at highest risk of contracting HIV infection. Applicants not belonging to the medical specialty were excluded.

A knowledge assessment questionnaire was developed by the panel of infection control experts with the help of HIV literature and previous studies [9, 10]. Another infection control expert reviewed the complete questionnaire and provided feedback on improvements. Each question was checked in terms of relevancy and clarity.

After written informed consent, study questionnaire was administered as pretest and after the symposium as posttest. The questionnaire was comprised of questions related to demographics, attitudes, awareness and knowledge related to HIV and PEP. The questionnaire was accessible both in English and Urdu and a time period of 20 min was given to complete the pretests. Speakers of the symposium delivered 30 min talk on the subject of HIV and PEP followed by questions and answers session. The total duration of the symposium was 3 h.

#### Sample size

There is no published literature available from Pakistan regarding HCPs’ knowledge of HIV and PEP. Therefore, with 95% confidence level, 80% power to detect a 20% increase in the score of an outcome of interest, with 2-sided alpha of 0.05, a final sample size of 165 study participants was determined.

#### Statistical analysis

Statistical Package for Social Sciences version 19 was used for analysis. Mean and standard deviation were computed for quantitative variable and frequency and percentage for qualitative variables. Frequency and percentages were analyzed of each item questions of awareness, attitude and knowledge scales and composite total score of knowledge items were reported as mean ± SD. Eleven knowledge based questions were recorded, with “Yes” (for correct answers; coded = 1) or “No” (for incorrect answers; coded = 0) response. McNemar test was used to see the association between pre and posttest variables and Bonferroni post hoc adjustment was used to detect the pretest and posttest significance. All P values were two-sided and considered as statistically significant if < 0.05.

### Results

Out of 391 participants attending the symposium, 365 consented to participate in the pre and posttest exercise. The demographic data is shown in Table 1. Majority of the participants belonged to private medical institutions (78%) and tertiary care centers (70.6%) with more female (56.7%) participation.

**Table 1:** Demographic characteristics of study population n = 365

	N (%)
Age, in years	28.1 ± 8.4
Median [IQR], range	26.5 [22–31], 16–64 years
Gender (n = 362)	
Male	158 (43.3)
Female	207 (56.7)
Type of institute (n = 365)	
Government	80 (21.9)
Private	285 (78)
Level of institute (n = 364)	
Tertiary care	258 (70.6)
Secondary care	68 (18.6)
Primary	38 (10.4)
Specialty (n = 356)	
Anesthesia	39 (10.7)
Emergency medicine	57 (15.6)
Medicine	65 (17.8)
Surgery	47 (12.9)
Students	89 (24.4)
Nursing staff	40 (11)
Others	19 (5.2)
Designation (n = 365)	
Doctor	85 (23.3)
Nurses/paramedics	178 (48.8)
Student	95 (26.7)
Others	7 (1.9)
Work experience (n = 362)	
6 month–2 years	97 (26.6)
3–5 years	82 (22.5)
6–8 years	38 (10.4)
> 8 years	64 (17.5)
Not applicable for student	81 (22.2)

Regarding awareness about HIV, 148 (40%) participants had previously received some information regarding HIV, almost half of the participants (n = 213, 58%) were aware of the term “post exposure prophylaxis” and few study participants (n = 52, 14.2%) attended any training related to PEP. There was no significant difference related to gender, type and level of institution, specialty, designation and work experience in terms of participants’ awareness with the term PEP or their attending any previous awareness session or training related to PEP.

Comparison of attitude of study participants regarding the importance of PEP is shown in Table 2. There was an overall statistically significant (P value < 0.001) improvement between pre and post symposium in the attitude of the participants. Majority of the change was observed among the participants who responded “don’t know” in the pretest and later agreed on the importance of PEP in posttest after the symposium.

**Table 2:** Comparison of attitude level before and after the symposium about post exposure prophylaxis (PEP)

	Before symposium n (%)	After symposium n (%)	McNemar’s test	P value
PEP is important for primary prevention				
Agree	286 (78.4)	325 (89.7)	35.99	< 0.001
Disagree	37 (10.1)	31 (8.6)		
Don’t know	42 (11.5)	6 (1.7)		
Training of PEP important for change in clinical practice				
Agree	329 (90.1)	354 (98)	24.15	< 0.001
Disagree	6 (1.6)	2 (0.6)		
Don’t know	30 (8.2)	5 (1.4)		
There should be PEP guideline in work areas				
Agree	340 (93.2)	352 (97.2)	14.33	0.001
Disagree	7 (1.9)	8 (2.2)		
Don’t know	18 (4.9)	2 (0.6)		
PEP reduce likelihood of being HIV positive				
Agree	290 (79.5)	339 (94.2)	51.28	< 0.001
Disagree	15 (4.1)	16 (4.4)		
Don’t know	60 (16.4)	5 (1.4)		
PEP is indicated for sharp injuries during patient management				
Agree	247 (67.7)	308 (84.9)	57.02	< 0.001
Disagree	41 (11.2)	44 (12.1)		
Don’t know agree	77 (21.1)	11 (3.0)		

The comparison of knowledge pre and post symposium is shown in Table 3. A statistically positive improvement in the number of participants giving correct answer was observed in 9 out of 11 questions (P value < 0.001). However, in question number 9 asking “by what ways HIV can be spread through contaminated needles?”, there was a decrease in number of participants giving correct answer but was not statistically significant. In question number 6 asking “by which route HIV is commonly transmitted from an infected person?”, there was no improvement in the knowledge post symposium as already the participants had a good knowledge about it, as 96.4% of participants gave a correct answer in the pretest and it increased to 98.4% posttest after the symposium with only 2% change which was not statistically significant. In question number 7 asking “When standard precautions should be taken by health care workers or providers?” around half of the participants knew the correct answer which did not change after the symposium.

**Table 3:** Pre and post symposium knowledge of HIV exposure among health workers (n = 365)

Questions related to knowledge		Percentage of participants with correct answers about HIV(n) (total number = 365)		% increase	P value
		Pre symposium n (%)	Post symposium n (%)		
1.	When PEP is indicated?	105 (28.8)	157 (43)	49.3	0.001*
2.	Whom to contact when exposure occurs?	85 (23.3)	234 (64.1)	175.1	0.001*
3.	What is the preferable time to take PEP after exposure?	202 (55.3)	259 (71.0)	28.4	0.001*
4.	What is effectiveness of PEP in terms of percentage in preventing HIV?	143 (40.3)	187 (51.2)	27.0	0.002*
5.	For how many days PEP should be taken after exposure?	117 (32.1)	267 (73.2)	128	0.001*
6.	By which route HIV is commonly transmitted from an infected person?	352 (96.4)	359 (98.4)	2.1	0.16
7.	When standard precautions should be taken by health care workers providers?	198 (54.2)	199 (54.5)	0.6	0.99
8.	People living with AIDS patients should avoid which of the following things?	328 (89.9)	342 (93.7)	4.2	0.02*
9.	By what ways HIV can be spread through contaminated needles	306 (83.8)	298 (81.6)	− 2.62	0.37
10.	If an HIV mother had emergency delivery and she did not receive HIV prophylaxis what is the chance in percentage of child getting the disease?	(9.6)	(57.8)	502.1	0.001*
11.	Prevalence of HIV infection in Pakistan	148 (40.5)	343 (94)	132.1	0.001*

*Significant values

The mean score of the participants before starting the intervention was 6.44 ± 1.84; median [IQR]; 6 [5–8] and it improved to 8.82 ± 2.17; median [IQR]; 9 [8–10] (out of 11 items) at the end of the symposium.

### Discussion

This study revealed gaps in the knowledge of HCPs about HIV as only 14% received any teaching and training on prevention of occupational exposure to HIV, which reflects lack of infrastructure at all levels to deal with communicable diseases in healthcare facilities in a developing country.

In addition, this study revealed that 42% of the participating HCPs never heard the term “post exposure prophylaxis” and only 28.8% had knowledge related to the indication of PEP, which is lower than quoted in other studies [11, 12]. Similarly, studies conducted in Tanzania, Kathmandu, Malaysia, Uganda and India indicated that health care workers have fair to poor knowledge about PEP [13–18]. This is an alarming situation as optimal post exposure care including the administration of antiretroviral drugs is an important step in prevention of HIV after accidental exposure [19]. The delay in the start of PEP not only increases the risk for the individuals to develop HIV infection, but also make these HCPs a potential source of HIV transmission [19].

Only 23.3% of the participants of the study knew the name of the person or office, which needs to be contacted after the accidental HIV exposure. This finding is similar to the results from the previous study conducted in Tanzania [20]. Not knowing the right contact person can lead to failure to report the event and delay in the start of treatment [21].

Only 32.1% of the participants knew the duration of treatment after HIV exposure. Previous studies have shown an impact of lack of knowledge on people completing their drugs regimen which requires 28 days [11, 13].

A statistically significant increase in the knowledge and a positive change in the attitude regarding PEP were observed among the participants who responded “don’t know” in the pretest and later agreed on the importance of PEP in posttest after the symposium. This indicates that education is a worthy effort in improving both knowledge and attitudes of HCPs.

Half of the participants had correct pre and post answers regarding the standard precautions that should be taken for all patients regardless of HIV. However previous studies have shown low compliance of HCPs towards the use of standard precaution with lack of time as the common reason [22].

Lack of awareness regarding the risk of transmission from an HIV-positive mother to her child in the absence of any intervention was observed, as only 9.6% of the participants could correctly answer; which is consistent with the previous literature [23]. This knowledge is however, significantly improved after the awareness session.

Majority of the participants (96.4%) had good base line knowledge regarding the route of transmission of HIV infection. However, a study done in rural India, quoted that only 25% of the participating HCPs knew the correct answer on routes of HIV transmission [24]. This dissimilarity could be due to the difference in the rural and urban populations of HCPs belonging to developing countries.

With the rise in HIV infected cases, knowledge of prevalence of a disease in the country is important to have certain level of suspicion index when dealing with patients. We observed an increase in the percentage of HCPs from 40.5 to 94% giving correct answers on prevalence after the symposium.

The strength of our study is that it not only assessed the baseline knowledge of the HCPs but it also assessed the change in knowledge after the educational symposium. This can help the medical facilities and institutions from developing countries to design an educational symposium on similar outlines provided in this study.

### Conclusion

Developing countries suffer from lack of infrastructure to provide basic level of education regarding prevention of occupational exposure to HCPs. Awareness symposium on regular basis can help in attaining basic knowledge among HCPs. There is also a need to implement standard prevention policies in health care facilities with regular audits and follow-ups.

### Limitations

There was no control group (without intervention) which may limit the ability to conclude the observed intervention effect. Secondly testing knowledge soon after the symposium cannot ensure long term retention of information. Therefore, there is a plan to hold regular awareness symposiums where previous participants can be evaluated for the retention of knowledge.

## Abbreviations

HCPs: health care providers
HIV: human immunodeficiency virus
PEP: post exposure
PMDC: Prophylaxis Medical and Dental Council
CME: continuing medical education
